# Social Isolation Modulates CLOCK Protein and Beta-Catenin Expression Pattern in Gonadotropin-Inhibitory Hormone Neurons in Male Rats

**DOI:** 10.3389/fendo.2017.00225

**Published:** 2017-09-07

**Authors:** Chuin Hau Teo, Tomoko Soga, Ishwar S. Parhar

**Affiliations:** ^1^School of Medicine and Health Sciences, Brain Research Institute, Monash University Malaysia, Subang Jaya, Malaysia

**Keywords:** stress, dorsomedial hypothalamus, gonadotropin-releasing hormone, reproduction, diurnal rhythmicity

## Abstract

Postweaning social isolation reduces the amplitude of the daily variation of CLOCK protein in the brain and induces lower reproductive activity. Gonadotropin-inhibitory hormone (GnIH) acts as an inhibitor in the reproductive system and has been linked to stress. Social isolation has been shown to lower neuronal activity of GnIH-expressing neurons in the dorsomedial hypothalamus (DMH). The exact mechanism by which social isolation may affect GnIH is still unclear. We investigated the impact of social isolation on regulatory cellular mechanisms in GnIH neurons. We examined *via* immunohistochemistry the expression of CLOCK protein at four different times throughout the day in GnIH cells tagged with enhanced fluorescent green protein (EGFP-GnIH) in 9-week-old adult male rats that have been raised for 6 weeks under postweaning social isolation and compared them with group-raised control rats of the same age. We also studied the expression of β-catenin—which has been shown to be affected by circadian proteins such as Bmal1—in EGFP-GnIH neurons to determine whether it could play a role in linking CLOCK in GnIH neurons. We found that social isolation modifies the pattern of CLOCK expression in GnIH neurons in the DMH. Socially isolated rats displayed greater CLOCK expression in the dark phase, while control rats displayed increased CLOCK expression in the light phase. Furthermore, β-catenin expression pattern in GnIH cells was disrupted by social isolation. This suggests that social isolation triggers changes in CLOCK and GnIH expression, which may be associated with an increase in nuclear β-catenin during the dark phase.

## Introduction

Social isolation is defined as a state of being where individuals are unable to contact or communicate other members of their community, whether by choice or by circumstance. Social isolation impacts many different species, but detrimental influence is more clearly observed on communal-minded organisms such as humans. Indeed, a 50% increase in the mortality rate has been observed in socially isolated individuals (1, 2). Severe social withdrawal is a rising phenomenon amongst youths (3) who are subsequently diagnosed with mood disorders including depression (4). Physiologically, social isolation beginning from the age of 5 in children may lead to greater mental difficulties (5).

Animal studies have shown the effect of social isolation on various neuronal systems in the rat brain, such as a dysregulated hypothalamic–pituitary–gonadal (HPG) axis (6–8), which results in sexual dysfunction.

In the HPG axis, gonadotropin-inhibitory hormone (GnIH) acts as an inhibitor of gonadotropin-releasing hormone (GnRH) and gonadotropins in mammals including humans (9–13). Discovered from the Japanese quail (14), GnIH is linked to stress-related disorder *via* the hypothalamic–pituitary–adrenal (HPA) axis. Stressful stimuli would induce GnIH neurons in the dorsomedial hypothalamus (DMH) to increase expression of c-Fos, indicating increased activity within those neurons when exposed to stress (15). The presence of glucocorticoid receptors, the major actors of the HPA axis, has been shown on GnIH neurons (16). Glucocorticoids have been shown to enhance transcription of GnIH mRNA (17, 18) and immunostained GnIH cells (19). In addition, social isolation impairs negative feedback regulation of the HPA axis (20) and fail to suppress corticosterone responses under acute stress (21). It is possible that social isolation increases sensitivity to stressful stimuli, thus making it easier for heightened glucocorticoid levels to induce an increase in GnIH mRNA transcription. However, the cellular mechanisms underlying social isolation-related changes in GnIH neurons remain to be fully elucidated.

In a previous study, alteration of the serotonergic system in the dorsal raphe of socially isolated rats caused an increase in serotonin receptors in GnIH (22) and serotonin fibers in GnIH neurons (23), suggesting serotonergic regulation of GnIH neurons and their possible involvement in the event of serotonergic dysfunctions such as major depressive disorder. C-fos expression in GnIH neurons was observed to be lower in cases of social isolation, indicating reduced GnIH neuronal activity (23). Socially isolated rats were also noted to behave in a more anxious manner while conducting an open field test during the dark phase (23). Social isolation can desynchronize diurnal rhythms (24), and a disruption of the day/night cycle has been indicated as both possible cause and effect of depression in humans (25). This disparity in behavior between light and dark phases suggest a possible diurnal component involved in the role of GnIH on social isolation-induced depressive behavior.

The serotonergic system is related to phase shifts in the circadian clock (26, 27), and so it is possible that social isolation-induced serotonergic dysfunction may result in circadian alterations to GnIH activity. CLOCK is a vital circadian protein that forms a heterodimer with another circadian protein, BMAL1, before binding to an E-box (CACGTG) site on a gene’s promoter region where it acts as a transcription factor (28). CLOCK has demonstrated rhythmic circadian oscillation, while BMAL1 does not within the DMH (29). This suggests that DMH neurons are regulated by circadian rhythm *via* clock expression. To investigate the possibility of a diurnal cycle existing within the rat GnIH neurons of the DMH, we focused on the CLOCK protein. GnIH mRNA expression has been demonstrated to be sensitive to photoperiodism in a study involving long-day and short-day photoperiods (30, 31). GnIH neurons of female rats have also demonstrated positive receptivity to melatonin, an important factor in the circadian clock (32, 33).

From a circadian perspective, β-catenin has also been associated with the circadian system in several ways, as it can alter circadian clock gene expression by inducing PER2 degradation (34), which in turn prevents the inhibition of the CLOCK-BMAL1 complex (35). β-catenin has a dual function as both adhesion protein and in gene transcription and is an important component of the canonical Wnt signaling pathway (36–38). In the cell, β-catenin presence is regulated by Wnt; in the absence of Wnt, β-catenin is targeted by GSK-3β for degradation, but the activation of the signaling pathway by Wnt reduces β-catenin degradation, causing it to accumulate and translocate into the nucleus where it activates TCF/LEF1 transcription factors that bind to Wnt target genes (39). β-catenin is also a protein that has been suggested to play a role in the molecular pathophysiology of stress, as sufferers of major depressive disorder exhibit decreased β-catenin mRNA levels in the prefrontal cortex (40). More importantly, the presence of β-catenin in the nucleus accumbens has been shown to reduce susceptibility to social isolation-induced depression in rodents *via* mediating an increase in the production of microRNAs related to stress resilience (41).

There is an emerging view that GnIH neuronal activity may be influenced by social isolation and subsequently play a role in the display of social isolation-related sexual dysfunction and stress-related physiological changes. This has led us to investigate whether a day/night cycle is a component of that role and to seek out potential models for GnIH activity that may involve diurnal action. First, we asked whether CLOCK protein is present in GnIH neurons and whether it exists, whether its expression is influenced by the day/night cycle. We then examined the effect of social isolation on the expression of CLOCK protein in GnIH neurons of the DMH. Next, as a potential link for CLOCK in GnIH neurons, we explored whether β-catenin is expressed in GnIH neurons and whether both social isolation and diurnal phases affect that expression. We also studied the effect of β-catenin activity on GnIH neuronal activity.

## Materials and Methods

### Animals and Housing Conditions

EGFP-GnIH transgenic rats (42) were randomly allocated to group-housing conditions (2–3 males/cage, *n* = 32) or isolated conditions (1 male/cage, *n* = 32). The grouped rats were housed in standard cages (dimensions: 276 mm × 445 mm × 204 mm, CLEA Japan, Inc., Tokyo, Japan), while isolated rats were housed in single cages (dimensions: 225 mm × 338 mm × 140 mm, CLEA Japan, Inc., Tokyo, Japan) that were wrapped in aluminum foil on all sides to prevent visual contact with other cages. Each single cage was placed on a separate shelf of an animal cage shelf with a blower unit attached to avoid olfactory social cues from other rats. Allocation of the rats was performed postweaning, 21 days after birth, and the rats were housed up till 9 weeks of age. The rats were reared under a 12-h light/dark cycle (lights on from 12:00 a.m. till 12:00 p.m.), and the temperature of the rooms were maintained at 22 ± 1°C and constant humidity for the duration of the housing prior to sampling. Food and water was made available *ad libitum* for the animals. Animal welfare and experimental ethics in BRIMS SPF animal facility were followed in line with the authorized guidelines laid out by Monash University Animal Ethics Community (MARP/2012/140, MARP/2017/021).

### Immunocytochemistry

Brain samples were collected at four periods in the day: 6:00 a.m. (ZT6), 12:00 p.m. (ZT12, commencement of the dark phase), 6:00 p.m. (ZT18) and 12:00 a.m. (ZT24, commencement of the light phase). The process was performed on male adult rats (*n* = 64, control: ZT6 *n* = 6, ZT12 *n* = 9, ZT18 *n* = 11, ZT24 *n* = 6; isolation: ZT6 *n* = 6, ZT12 *n* = 8, ZT18 *n* = 12, ZT24 *n* = 6, 9 weeks old) following the perfusion and fixation protocol that has been described in a previous experiment (42). Immunocytochemistry for CLOCK and β-catenin was performed on the DMH sections obtained through sectioning. Sectioning of the rat brain took place in a cryostat chamber (Leica CM1900, Leica Biosystems, Heidelberg, Germany) with its temperature set to −20°C. Each section was sliced along the coronal plane at a thickness of 30 µm, starting from the beginning till the end of the DMH (bregma −0.26 to −4.16 mm), with approximately 80 tissue sections obtained per brain. The sections were placed in an antifreeze solution (30% ethylene glycol, 20% glycerol in 0.1 M PB solution) and stored at −20°C in a 12-well plate. Alternating sections were divided and stored in separate wells to provide two sets of samples for analysis. The tissue sections were washed twice with ice-cold 0.1 M PBS, with each repetition incubated in an incubation chamber for 10 min at room temperature and gently shaken at 60 rpm. The washing step would be repeated in between each major incubation step of the procedure. The sections were then incubated in a blocking solution [40 µL normal goat serum (NGS), 10 µL 0.5% Triton-X and 1950 µL PBS in 2 mL/well for CLOCK, with the NGS being replaced by normal horse serum (NHS) for β-catenin] for 1 h in the same conditions as above. After washing, the sections were incubated with a rabbit anti-CLOCK antibody diluted 1:400 [5.0 µL clock antiserum (H-276) sc-25361, Santa Cruz Laboratories, USA; 40 µL NGS, 10 µL 0.5% Triton-X and 1945 µL 0.1 M PBS in 2 mL/well] or with a mouse anti-β-catenin antibody diluted 1:400 [beta-catenin antiserum (12F7), ab22656, Abcam Inc., MA, USA] for 1 h in the incubation chamber before being transferred to 4°C storage for overnight incubation of 24 h. The antibody immunogen sequences can be found in the supplementary material (Table S1 in Supplementary Material). Subsequently, the sections were incubated in the corresponding secondary antibody solution [10 µL biotinylated anti-rabbit immunoglobulin-G (IgG) for CLOCK, 40 µL NGS, 1,950 µL 0.1 M PBS in 2 mL/well and 10 µL biotinylated anti-mouse IgG for β-catenin, 40 µL NHS, 1,950 µL 0.1 M PBS in 2 mL/well (Vectastain ABC Elite kit, Vector Laboratories, Burlingame, CA, USA)] for 45 min, followed by incubation with A-B complex [40 µL avidin–biotinylated horseradish peroxidase complex (Vectastain ABC Elite kit, Vector Laboratories, Burlingame, CA, USA), 1,960 µL 0.1 M PBS in 2 mL/well] for 45 min and finally Streptavidin Alexa Fluor 594 [3.5 µL (S32356, Invitrogen Corporation, USA), 1,996.5 µL 0.1 M PBS in 2 mL/well] 30-min incubation for the purposes of visualization. The sections were pasted on microscope slides (Superfrost PLUS, Fisher Scientific, Pittsburgh, PA, USA) and mounted with VECTASHIELD Mounting Medium (Vector Laboratories, Burlingame, CA, USA) for fluorescence preservation before being stored at 4°C.

CLOCK and β-catenin antibody specificity was tested using a blank buffer test where one specimen would be incubated with primary antibody solution (1:400 clock antiserum or beta-catenin antiserum) (in 40 µL NGS, 10 µL 0.5% Triton-X, and 1,945 µL 0.1 M PBS in 2 mL/well), while the other specimen would have the primary antibody omitted (40 µL NGS, 10 µL 0.5% Triton-X, and 1,950 µL 0.1 M PBS in 2 mL/well), with the remainder of the steps as per the immunocytochemistry procedure detailed above (Figure S1 in Supplementary Material). The CLOCK antibody utilized in this experiment has been previously used successfully for other published papers (43, 44). The specificity of the β-catenin antibody was also tested by absorption tests (1:400) with human β-catenin peptide (5 µg/mL beta-catenin peptide, ab16377, Abcam Inc., MA, USA). Incubation of the β-catenin antibody with the β-catenin peptide inhibited the staining in the rat hypothalamus (Figure S1 in Supplementary Material).

### Cell Counting and Colocalization

The Leica laser-scanning microscope (Leica Microsystems, Germany) was used for counting of immunoreactive GnIH cells and determining immunoreactive clock and β-catenin activity in the fluorescent GnIH neurons. The EGFP and Alexa Fluor 594 fluorophores were excited by lasers emitting at wavelengths of 488 and 543.5 nm respectively. By using the Leica Application Suite X software, the Z-steps were obtained at an interval of 1.25 µm *via* depth scanning with the use of a 10× objective lens for CLOCK immunoreactive specimens and 20× objective lens for β-catenin immunoreactive specimens. A higher magnification was chosen for the latter to better distinguish the differences in β-catenin staining morphology. Maximum intensity projections were utilized to portray the full gamut of fluorophore expression in the sample. Each of the 1.25-µm Z-steps was examined carefully to confirm that the neurons counted were fully present in the observed section. All EGFP-GnIH neurons in the DMH region were counted. The presence of CLOCK or β-catenin (red fluorescence) in association with GnIH neurons (green fluorescence) were marked as colocalization, appearing yellow on the images captured, and the percentage of GnIH neurons expressing CLOCK or β-catenin were recorded. For β-catenin, colocalization is further divided into nuclear and cytoplasmic depending on the primary area of β-catenin localization observed. 51 samples were counted for CLOCK immunoreactivity (control: *n* = 24 and isolation: *n* = 27, *n* = 6 for each of control at ZT6, ZT12 and ZT18, and ZT24, and *n* = 6 for isolation at ZT6, *n* = 7 for isolation at ZT12, *n* = 8 for isolation at ZT18, and *n* = 6 for isolation at ZT24), and 30 samples were counted for β-catenin immunoreactivity (control: *n* = 15 and isolation: *n* = 15, *n* = 6 for each of control and isolation at ZT12, *n* = 9 for each of control and isolation at ZT18). Approximately 30 DMH sections were counted for each brain, with the total GnIH cells counted per brain approximating 1,000.

### EFGP-GnIH Intensity in GnIH Neurons

The intensity of each EGFP-GnIH-expressing neuron was measured using the inbuilt software of the Leica laser-scanning microscope (Leica Microsystems, Germany), using the same images that have been captured for the cell counting procedure above. Each EGFP-GnIH neuron was manually designated as a single region using the software. The software would then record the intensity value of the fluorescent green expressed by each neuron. The data were tabulated and manually analyzed to determine the average intensity of the GnIH neuron in the samples. The intensity value is measured as a grayscale number ranging from 0 to 255, with 0 being the darkest (black) and 255 being the brightest (white). It is defined as the average brightness of all the pixels in the selected region, which would constitute a single neuron. 12 samples were measured for EGFP-GnIH intensity (control: *n* = 6 and isolation: *n* = 6 at ZT18). The total number of GnIH cells counted per brain approximated 1,000.

### DAPI Staining

DAPI staining was carried out to confirm colocalization of β-catenin within the nucleus of the GnIH neurons. After completion of the Alexa Fluor 594 staining following the immunocytochemistry procedure as detailed above, the sections were then incubated for 15 min in DAPI dihydrochloride solution [2.5 µL DAPI dihydrochloride (28718-90-3, Sigma-Aldrich, MO, USA), 1,997.5 µL 0.1 M PBS in 2 mL/well] for nuclear staining before being pasted on slides as per the aforementioned protocol (DAPI-stained specimens, *n* = 2). The Eclipse 90i Nikon fluorescent microscope (Nikon Instruments, Tokyo, Japan) equipped with a Nikon DXM 1200C camera and NIS-Element 3.0 software was used to capture images of the β-catenin and DAPI-stained sections. The EGFP, Alexa Fluor 594, and DAPI fluorophores were excited by lasers emitting at wavelengths of 488, 543.5, and 358 nm, respectively. Fluorescent microscope images were captured at a resolution of 4,116 × 3,072 pixels and at a magnification of 20×. Each fluorophore was captured separately at the same focal point before being merged into a single picture using NIS-Element 3.0 software.

### Statistics

Data are presented as means ± SEM in all bar graphs. Immunohistochemistry and EGFP-GnIH intensity results were analyzed using two-way ANOVA to determine the significance of the effect of time, housing, and the interaction between the two, before applying *t*-tests for further analysis. The change in CLOCK expression across time points was analyzed by a univariate repeated measures test using SPSS 20 (IBM, Chicago, IL, USA). Significance was set as *p* < 0.05.

## Results

### CLOCK Expression in GnIH Neurons

**Figure 1:**
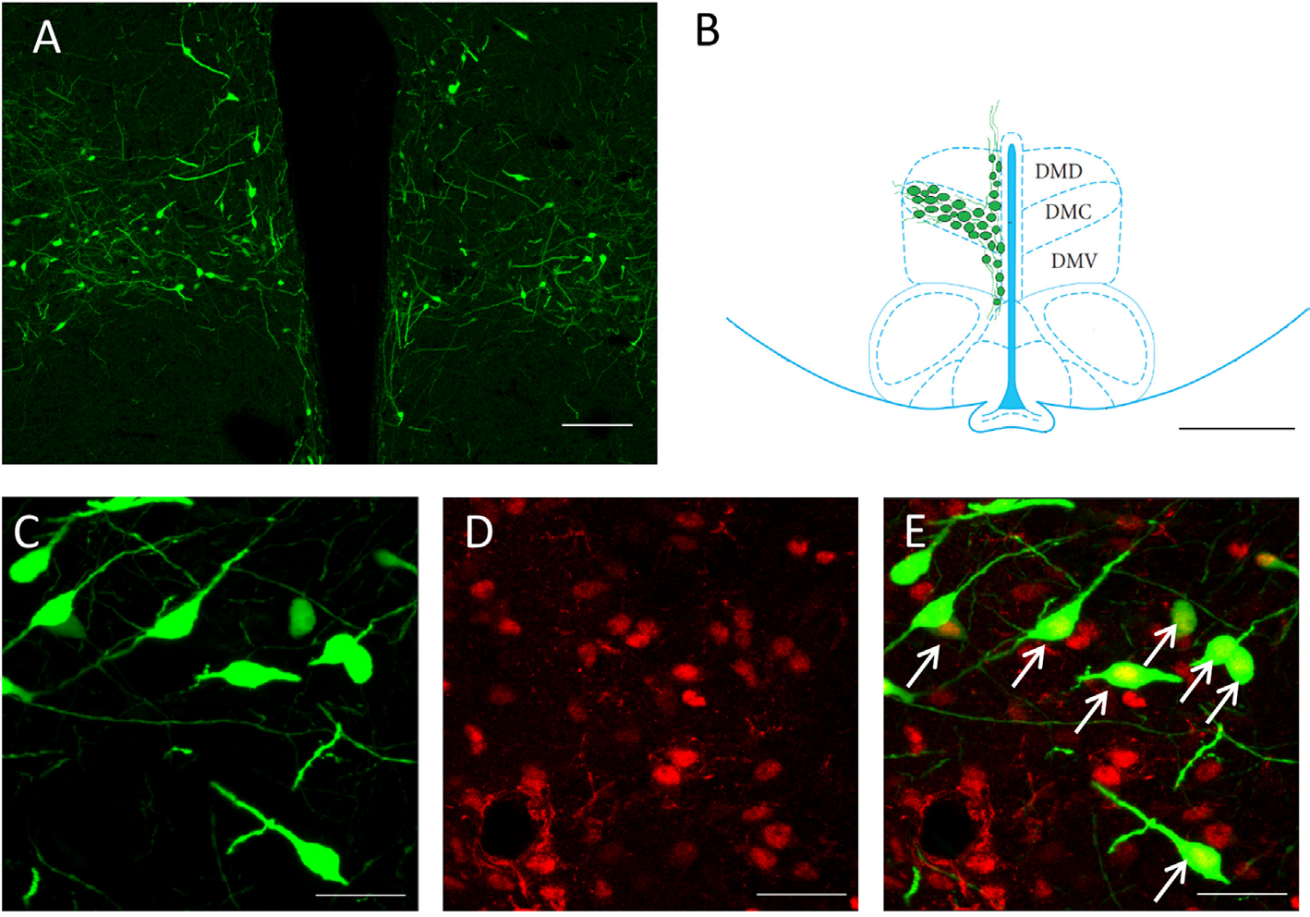
Expression of CLOCK protein within GnIH neurons in the DMH region. **(A)** An overview of GnIH-expressing neurons in the DMH at 10× magnification. **(B)** Primary area of GnIH neuron expression mapped out on the rat brain atlas. Bregma of image is at −3.12 mm. Sectioned areas were taken from a bregma of −0.26 to −4.16 mm. Scale bar = 100 µm **(A)** and 200 µm **(B)**. **(C)** EGFP-GnIH-expressing neurons (green), **(D)** CLOCK-immunostained neurons (red), and **(E)** combined EGFP-GnIH (green) and CLOCK (red) images at 40× magnification. Scale bar = 20 µm **(C–E)**. White arrows indicate the presence of CLOCK localization. DMC, dorsomedial hypothalamic nucleus, compact; DMD, dorsomedial hypothalamic nucleus, dorsal; DMV, dorsomedial hypothalamic nucleus; ventral; GnIH, gonadotropin-inhibitory hormone; DMH, dorsomedial hypothalamus.

To identify any possible effects of social isolation on CLOCK rhythms, we selected samples from four different time points; ZT6, ZT12 and ZT18, and ZT24 for our measurements. The primary focus for the cell counting was the GnIH population of the DMH region (Figures 1A,B). All GnIH neurons and GnIH neurons expressing CLOCK proteins were counted. CLOCK colocalization with GnIH neurons was observed in the nucleus of the cells (Figures 1C–E).

**Figure 2:**
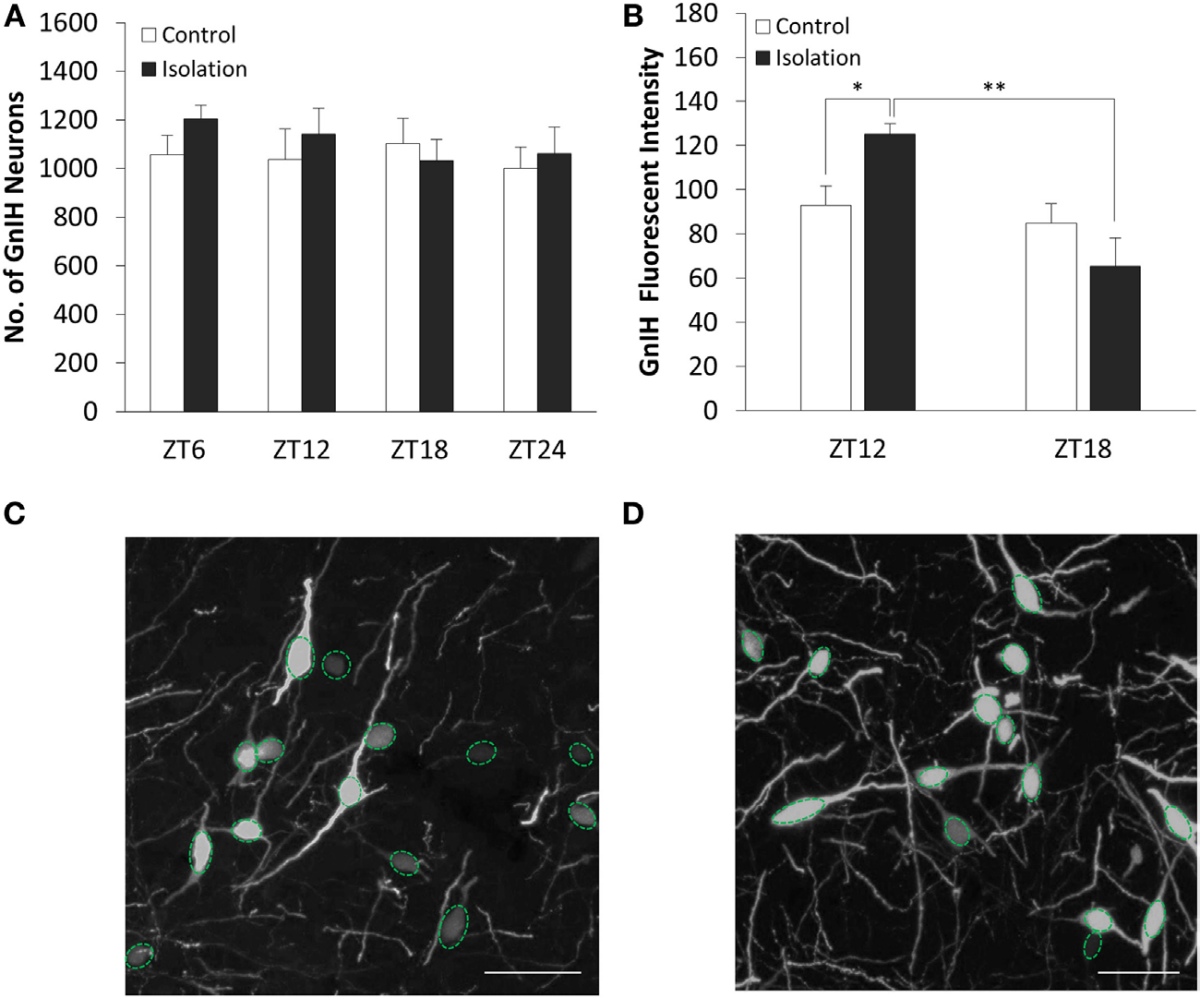
The effect of social isolation on CLOCK expression in gonadotropin-inhibitory hormone (GnIH) neurons in the dorsomedial hypothalamus (DMH) region. **(A)** Total GnIH cell numbers aggregated by ZT time, for control and isolated groups in the DMH (control: ZT6 *n* = 6, ZT12 *n* = 6, ZT18 *n* = 6, ZT24 *n* = 6 and isolation: ZT6 *n* = 6, ZT12 *n* = 7, ZT18 *n* = 8, ZT24 *n* = 6). **(B)** Average intensity of GnIH neurons of control and isolated rats at ZT12 and ZT18 (control: ZT12 *n* = 6, ZT18 *n* = 6 and isolation: ZT12 *n* = 6, ZT18 *n* = 6). **(C)** Image of DMH GnIH neurons at 40× magnification for control (ZT12) and **(D)** isolation (ZT12). Scale bar = 50 µm **(C,D)**.

The number of GnIH cells in the DMH did not vary significantly both between control and isolation and between time points (Figure 2A). We found that the interaction between housing and time was a significant factor in the change of GnIH neuronal fluorescent intensity, as was time itself (control: *n* = 12, isolation: *n* = 12, *F*[1, 24] = 13.61, *p* < 0.01). The average intensity of GnIH neurons was observed to be higher in socially isolated animals at ZT12 in comparison to control rats at the same time point [control ZT12: 92.94 ± 8.89 (*n* = 6) and isolation ZT12: 125.31 ± 4.61 (*n* = 6), *p* < 0.05; Figure 2B]. A significant difference was also observed comparing the intensity of isolated rats at ZT12 to similarly isolated rats at ZT18 [isolation ZT12: 125.31 ± 4.61 (*n* = 6) and isolation ZT18: 65.51 ± 12.62 (*n* = 6), *p* < 0.01; Figure 2B]. This difference in intensity can be observed in control (Figure 2C) and isolation groups (Figure 2D) in the light phase, with higher intensity for isolation groups. There was no significant difference to be found in intensity measurements for control rats at both time points.

**Figure 3:**
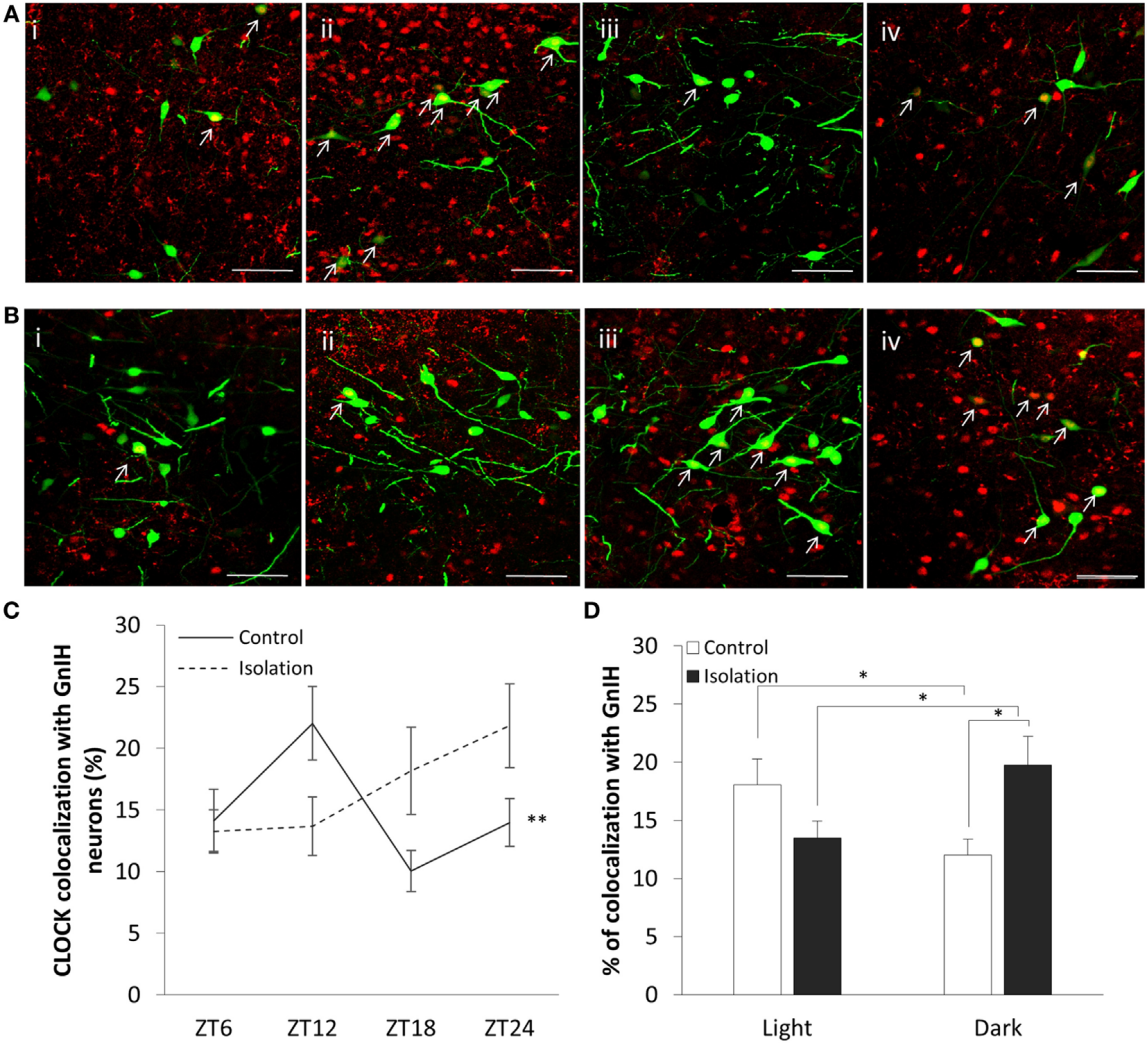
Colocalization of CLOCK protein within gonadotropin-inhibitory hormone (GnIH) neurons in the dorsomedial hypothalamus (DMH) region across different time points. **(A)** EGFP-GnIH neurons (green) and CLOCK-immunostaining (red) in the DMH of group-housed rats sampled at ZT6 (i), ZT12 (ii), ZT18 (iii), and ZT24 (iv). **(B)** EGFP-GnIH neurons (green) and CLOCK-immunostaining (red) in the DMH of isolation-housed rats sampled at ZT6, ZT12, ZT18, and ZT24. White arrows indicate colocalization between GnIH neurons and CLOCK protein. Scale bar = 50 µm. **(C)** Comparison of CLOCK colocalization percentage in the GnIH neurons of control and isolated rats over time (ZT 24 = lights on, ZT 12 = lights off, control: ZT6 *n* = 6, ZT12 *n* = 6, ZT18 *n* = 6, ZT24 *n* = 6 and isolation: ZT6 *n* = 6, ZT12 *n* = 7, ZT18 *n* = 8, ZT24 *n* = 6). **(D)** Comparison of CLOCK colocalization percentage in the GnIH neurons of control and isolated rats, grouped by light (ZT6 and ZT12) and dark (ZT18 and ZT24) phases (control: light *n* = 12, dark *n* = 12 and isolation: light *n* = 13, dark *n* = 14). Data are presented as means ± SEM for each set. Significance was set at *p* < 0.05.

The expression of CLOCK within the DMH, and its colocalization with GnIH neurons, appears to rise and fall as we travel across each time point in the control group (Figure 3A), starting at ZT6 (Figure 3A, i), peaking at ZT12 (Figure 3A, ii) before dropping down at ZT18 (Figure 3A, iii), and beginning to rise again by ZT24 (Figure 3A, iv). Within the isolated group, the changes in expression levels appear to increase (Figure 3B), with ZT6 (Figure 3B, i) and ZT12 demonstrating lower CLOCK levels than (Figure 3B, ii) ZT18 (Figure 3B, iii) and ZT24 (Figure 3B, iv).

The interaction between housing and time was indicated as a significant factor for the changes in colocalization of CLOCK and GnIH neurons throughout a 24-hour period (control: *n* = 24, isolation: *n* = 27, *F*[1, 51] = 4.25, *p* < 0.01). Using the repeated measures test, the percentage of expression of CLOCK protein in GnIH neurons for the control group demonstrated a significant fluctuation in values when comparing the difference between each of the time points (control: *F*[1,24] = 5.57, *p* < 0.01; Figure 3C), while any difference in CLOCK expression within the GnIH neurons from the isolated group was found to be insignificant when compared in the same fashion.

Subsequently, we grouped the specimens into light (ZT6 and ZT12) and dark (ZT18 and ZT24) phases and compared the colocalization between the two groups. We discovered a strong correlation between housing and phase interaction with CLOCK colocalization (control: *n* = 24, isolation: *n* = 27, *F*[1, 51] = 9.83, *p* < 0.01). CLOCK protein colocalization was significantly higher in the light phase compared to the dark phase for control group rats, while the reverse was observed by time for the isolation-housed rats [control ZT6 + ZT12: 18.09 ± 2.21 (*n* = 12) and control ZT18 + ZT24: 12.02 ± 1.36 (*n* = 12), *p* = 0.029; isolation ZT6 + ZT12: 13.49 ± 1.45 (*n* = 13) and isolation ZT18 + ZT24: 19.76 ± 2.44 (*n* = 14), *p* < 0.05; Figure 3D]. Comparing between groups, while we did not observe any significant differences between control and isolation in the light phase, isolated animals demonstrated higher colocalization for CLOCK in the dark phase [control ZT18 + ZT24: 12.02 ± 1.35 (*n* = 12) and isolation ZT18 + ZT24: 19.76 ± 2.45 (*n* = 14), *p* < 0.05; Figure 3D].

### β-Catenin Expression in GnIH Neurons

**Figure 4:**
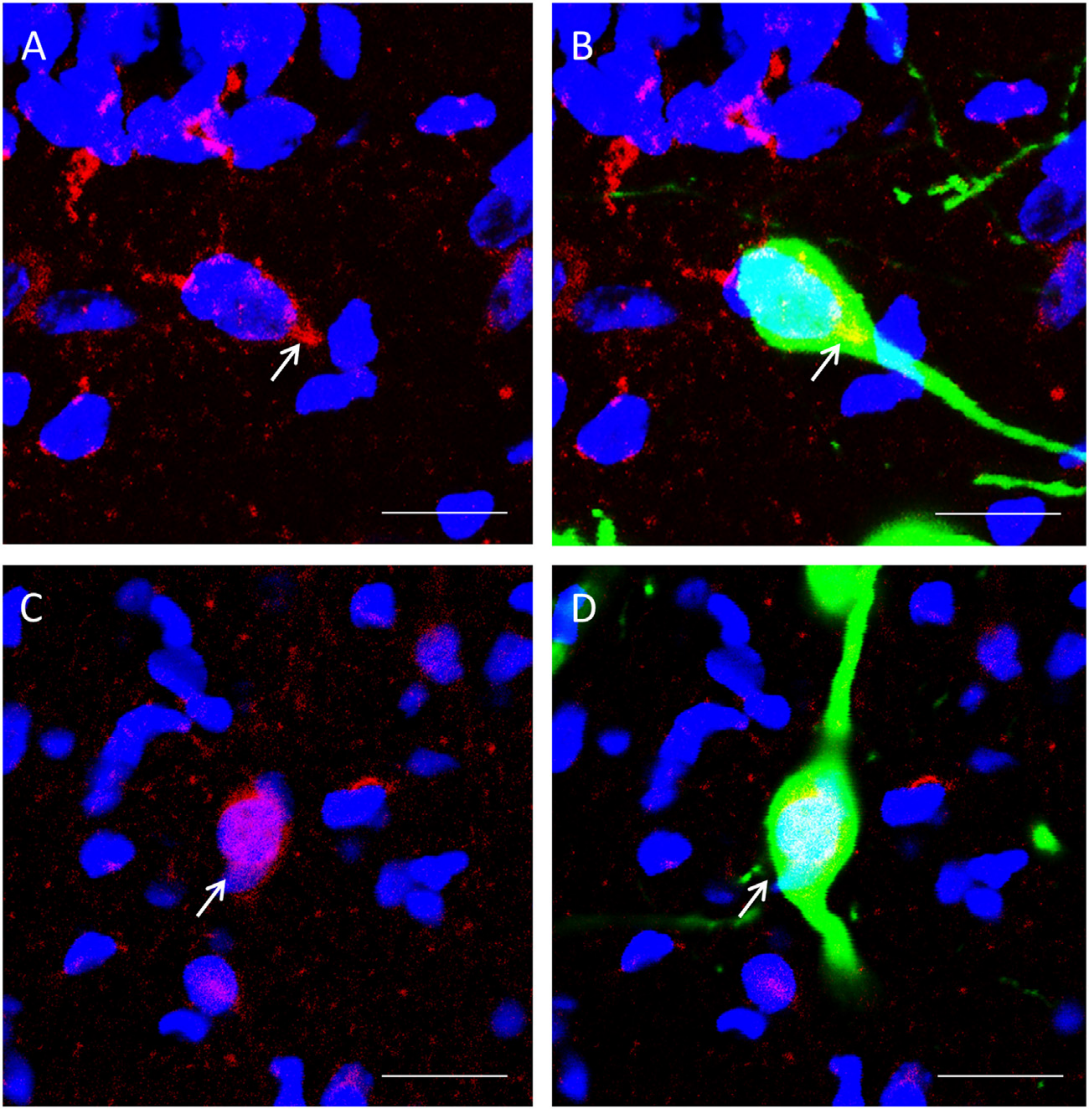
The colocalization of β-catenin immunostaining with DAPI within gonadotropin-inhibitory hormone (GnIH) neurons in the dorsomedial hypothalamus (DMH) region. **(A)** β-catenin immunostaining (red) and DAPI cytoplasmic staining (blue) and **(B)** colocalization with GnIH neuron (green). **(C)** β-catenin immunostaining (red) in the cytoplasm with DAPI nuclear staining (blue) and **(D)** colocalization with GnIH neuron (green). Scale bar = 20 µm. White arrows indicate the presence of β-catenin colocalization with GnIH neurons.

β-catenin localization could be observed in GnIH neurons. We spotted two distinct morphologies; one where colocalization staining of β-catenin and nuclear DAPI staining expression of β-catenin within the cell demonstrated staining of the cytoplasm (Figures 4A,B) and one where β-catenin is localized primarily in the nucleus (Figures 4C,D).

**Figure 5:**
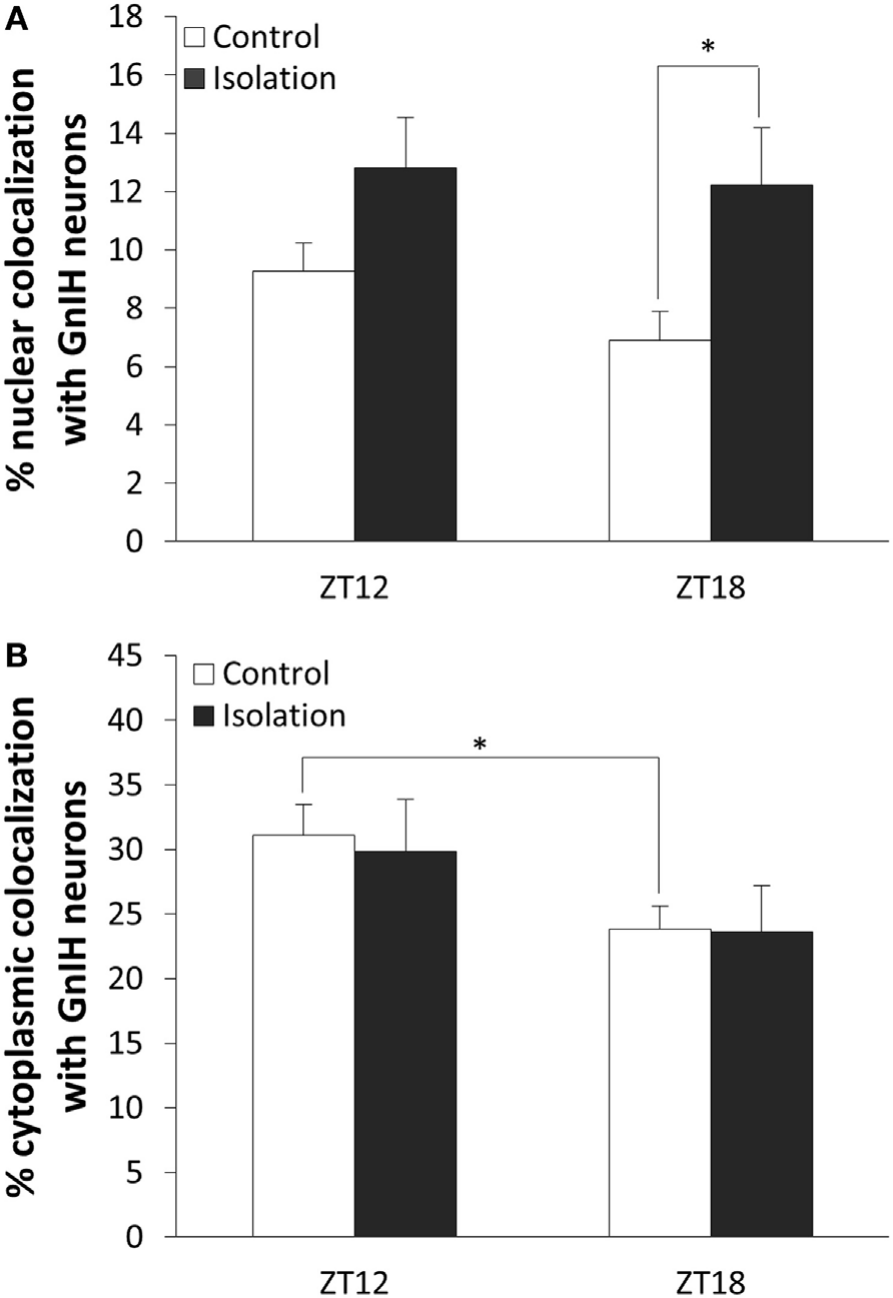
The effect of postweaning social isolation on β-catenin colocalization with gonadotropin-inhibitory hormone (GnIH) neurons in the dorsomedial hypothalamus region. **(A)** Comparison of β-catenin nuclear colocalization within GnIH neurons of the control and isolated rats at ZT12 and ZT18 (control: *n* = 6 and isolation: *n* = 6 for ZT12 and control: *n* = 9 and isolation: *n* = 9 for ZT18). **(B)** Comparison of β-catenin cytoplasmic colocalization in GnIH neurons of control and isolated rats in ZT12 and ZT18 (control: *n* = 6 and isolation: *n* = 6 for ZT12 and control: *n* = 9 and isolation: *n* = 9 for ZT18). Data are presented as means ± SEM for each set. Significance was set at *p* < 0.05.

To determine whether β-catenin expression in GnIH can be influenced by temporal and housing factors, we selected specimens from ZT12 and ZT18, so chosen because they represented the highest and lowest points in CLOCK expression as observed earlier. We also focused on neurons where β-catenin is co-localized in the nucleus as an indicator of the protein’s activity in its role as a transcription factor. Analysis of the data of cytoplasmic colocalization of β-catenin pointed to time points as the influencing factor (control: *n* = 15, isolation: *n* = 15, *F*[1, 30] = 4.70, *p* < 0.05). Control-housed specimens demonstrated higher cytoplasmic colocalization of β-catenin when comparing ZT12 to ZT18 [control ZT12: 31.10 ± 2.35 (*n* = 6) and control ZT18: 23.83 ± 1.79 (*n* = 9), *p* < 0.05; Figure 5A]. We also observed that nuclear colocalization was conversely affected by housing and not time points (control: *n* = 15, isolation: *n* = 15, *F*[1, 30] = 7.75, *p* < 0.01), and we were able to identify a significant elevation in nuclear colocalization for isolated rats in ZT18 compared to the control [control ZT18: 6.91 ± 0.99 (*n* = 9) and isolation ZT18: 12.22 ± 1.98 (*n* = 9), *p* < 0.05; Figure 5B]. No difference was observed for the GnIH neurons of isolated rats.

**Figure 6:**
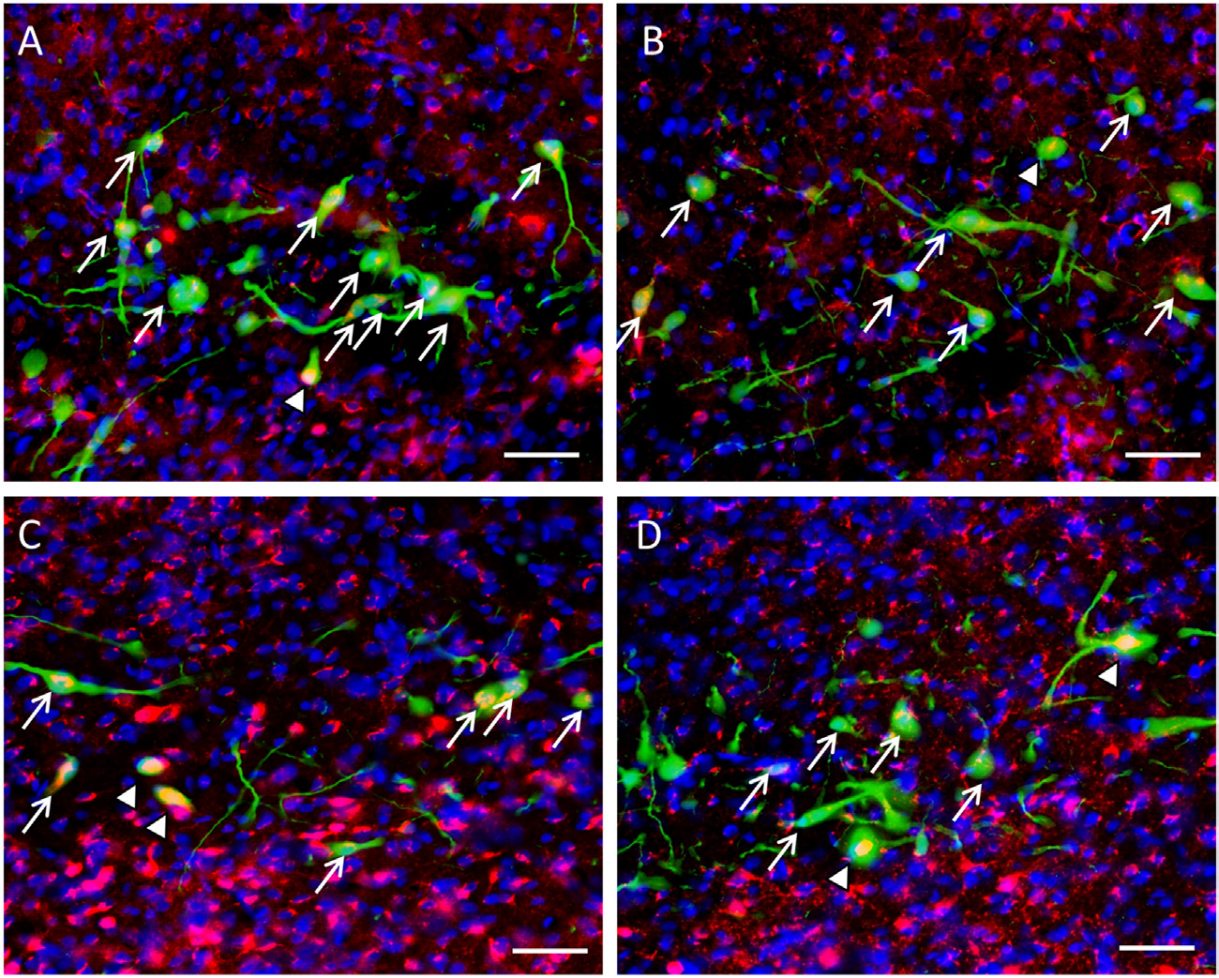
Colocalization of β-catenin within gonadotropin-inhibitory hormone (GnIH) neurons in the dorsomedial hypothalamus (DMH) region across different time points. β-catenin immunostaining (red) was observed within GnIH neurons (green) in **(A)** control at ZT12, **(B)** control at ZT18, **(C)** isolation at ZT12, and **(D)** isolation at ZT18. Scale bar = 25 µm. White arrows indicate cytoplasmic colocalization, while white arrowheads indicate colocalization within or around the nucleus of GnIH neurons.

Expression of β-catenin within the nuclei of GnIH neurons in control rats (Figures 6A,B) was less prevalent than isolated rats in ZT18 (Figures 6C,D).

## Discussion

In this study, we observed daily variation of CLOCK expression in GnIH neurons. Compared to the control group, which displayed a fluctuation in CLOCK levels throughout the day, the isolated animals did not exhibit significant difference in CLOCK expression. CLOCK expression was significantly heightened in the dark phase in isolated rats compared to control rats. Looking at β-catenin, we found that they displayed a difference between ZT12 and ZT18 in both cytoplasmic and overall colocalization, increasing at ZT12 and decreasing at ZT18. We also observed higher EGFP intensity in isolated rats compared to control rats at ZT12.

### CLOCK Expression in GnIH Neurons

CLOCK protein was observed within GnIH neurons in the DMH. Counting only the CLOCK-positive neurons that were also co-localized with GnIH expression, CLOCK was found to be expressed more highly in the light phase in control animals and more highly in the dark phase for isolated animals. Isolated animals also saw reduced differences in CLOCK expression between each time point measured.

In control rats, the peak of CLOCK expression was in the light phase compared to the dark, which supports the findings of Wyse and Coogan (29) where they determined the rhythmic nature of CLOCK in the DMH. Prior studies have demonstrated fluctuations in GnIH peptide and mRNA levels according to photoperiods, demonstrating a link between GnIH and the diurnal cycle in multiple species (30, 32, 45). A recent study performed on Syrian hamsters demonstrated differential action of vasointestinal peptide on GnIH cellular activity depending on the time of day (33). The same study also observed a rhythmic expression of PER1 protein in GnIH neurons (33), supporting our findings that GnIH neurons maintain their own internal clock. The presence of CLOCK and its rhythmicity suggests that the functionality of GnIH neurons in the DMH may be influenced by circadian rhythms.

In particular, the observed reduction of differences in CLOCK expression across time in GnIH cells in isolated animals leads us to view the impact of social isolation as weakening the cyclic pattern of CLOCK expression. This is similar to a symptom of depression that has been observed in humans; a comprehensive postmortem study was conducted on patients suffering from major depressive disorder, discovering that circadian genes in depressed patients displayed a weaker cyclic pattern and that the expression of those genes exhibited less synchronicity between each other—in the normal scheme of things, the rise of a set of cyclic circadian genes would be linked to the fall of another set of circadian genes, but in depressed patients, the expression of the genes lose that synchronicity (46). In our study, we observed that the expression of CLOCK is heightened in the dark phase for isolated animals, in relative terms, compared to the regular CLOCK expression in control animals. Previous behavior experiments have indicated a more pronounced effect of social isolation on anxiety-like behavior in the dark (23). As those behaviors are linked to GnIH activity, the desynchronicity of CLOCK and its relative overexpression during the dark phase may be a factor. However, the cell counts performed show that GnIH neurons do not observably increase in number, which indicates that this link may be through the neuronal activity of existing neurons or the release of GnIH to related areas to control anxiety, rather than proliferation of GnIH neurons.

As EGFP is tagged to the GnIH promoter (42), increased activation of the promoter would result in a higher intensity value. Isolated rats displayed elevated intensity of GnIH neurons at ZT12 in comparison to their control counterparts. This indicates that the isolation process may have introduced a change in the basal activity patterns of GnIH neurons in the DMH, with increased activity during the light phase and reduced activity during the dark. The correlation appears to be inverse to that of the pattern observed in CLOCK, whereas social isolation has reduced the variation in CLOCK expression in GnIH neurons, it has also induced a variation in GnIH promoter activity across light and dark phases.

As under social isolation, both GnIH and GnRH expressions are reduced (23), it is likely GnIH activity is normally low since it acts mainly as a counter measure to GnRH activity. We conjecture that while social isolation may exert a suppressive effect on GnIH neurons at a basal level, it also renders them more sensitive to stressful stimuli, causing them to react in a pronounced way upon exposure to stress (21).

### β-Catenin Expression in GnIH Neurons

This study is the first to observe the expression of β-catenin within GnIH neurons. Between 30 and 40% of GnIH neurons counted expressed observable levels of β-catenin, and different morphologies of β-catenin staining was observed in the cells, where it may be present in the cytoplasm, where the amount of β-catenin in the cytoplasm is in the process of increasing until it has saturated the binding sites, or the nucleus, where β-catenin has translocated from the saturated cytoplasm into the nucleus. This appears to match the role of β-catenin in the Wnt signaling pathway; namely its accumulation in the cytoplasm and subsequent translocation into the nucleus. β-catenin’s presence in the nucleus would indicate its activity as a transcription factor. This would further suggest that β-catenin and the Wnt signaling pathway is active within GnIH neurons.

Nuclear β-catenin levels in GnIH neurons in the dark phase for isolation-raised rats are increased over that of the control group. This appears to suggest that social isolation may elevate β-catenin activity particularly in its role as a nuclear transcription factor. It is also possible that expression of β-catenin, at least within the DMH, may change according to the time of the day. In accordance with that, we found that in the control group, β-catenin expression in the cytoplasm was observed to fluctuate according to the phase, with a higher expression at ZT12 compared to ZT18. These time points match the peak and trough of CLOCK expression in the control group. In the isolated group, this fluctuation appears to be reduced, similarly to CLOCK under the same circumstances. This suggests a possible link between CLOCK and β-catenin expression. It also suggests that the Wnt signaling pathway may be influenced by the circadian cycle, as an increase of β-catenin levels in the cytoplasm is a prerequisite step for nuclear translocation. It may be possible that the heightened nuclear β-catenin levels in isolated rats during the dark phase may correspond to the increased CLOCK expression levels that we have described in the results section pertaining to CLOCK (Figure 3D).

β-catenin cytoplasmic colocalization varies according to the time of day, which points to the presence of a diurnal component in the potential interactions of β-catenin in GnIH neurons. The altered light/dark phase of CLOCK may affect the expression of β-catenin.

The relation between CLOCK and β-catenin in neuronal cells has not been elucidated by any other studies. Given that β-catenin has been predicted to have an E-box (CACGTG) binding site on its promoter, it is certainly possible that CLOCK may drive β-catenin expression directly *via* binding to the E-box in liver cells (47). This appears to explain the reduction of β-catenin fluctuations in relation to the reduction of CLOCK fluctuations as observed in our results, as well as the increase in CLOCK expression corresponding to a rise in nuclear β-catenin levels.

The potential link between β-catenin and GnIH neuronal activity appears to be clearer. Although the GnIH promoter is predicted to express a binding site for TCF/LEF1, the reduced neuronal activity in conjunction with increased β-catenin activity makes it less likely that β-catenin directly promotes activation of the GnIH gene *via* the Wnt signaling pathway. In their article regarding the role of β-catenin in preventing depression, Dias et al. (41) made specific mention of β-catenin’s control of Dicer1, a gene highly involved in the production of microRNAs as a factor in stress resilience. Similarly microRNAs may also play a part in β-catenin’s role within GnIH neurons. Two microRNAs, mir-155 and mir-7b, in particular, have been shown to decrease c-Fos protein levels by suppressing the translation of its mRNA transcript into protein (48, 49). Both microRNAs are regulated by Dicer1.

It is possible that the increased expression of β-catenin in isolated rats is related to decreased GnIH neuronal activity through this pathway. Expression of nuclear β-catenin may in this way increase the expression of Dicer1, which in turn promotes the production of microRNAs that reduce GnIH neuronal activity through the suppression of c-Fos translational activity. Alterations of β-catenin activity in the brain in response to long-term physiological changes have been observed elsewhere, notably in aging, where nuclear β-catenin was increased with age and demonstrated a resistive effect against age-related neural degeneration (50). These findings indicate that β-catenin plays a role in neuro protection. We theorize that the change in nuclear β-catenin expression patterns within GnIH neurons in the DMH under isolation conditions suggests the presence of a possible response mechanism to chronic stress, which promotes translocation of β-catenin into the nucleus as a neuroprotective measure.

**Figure 7:**
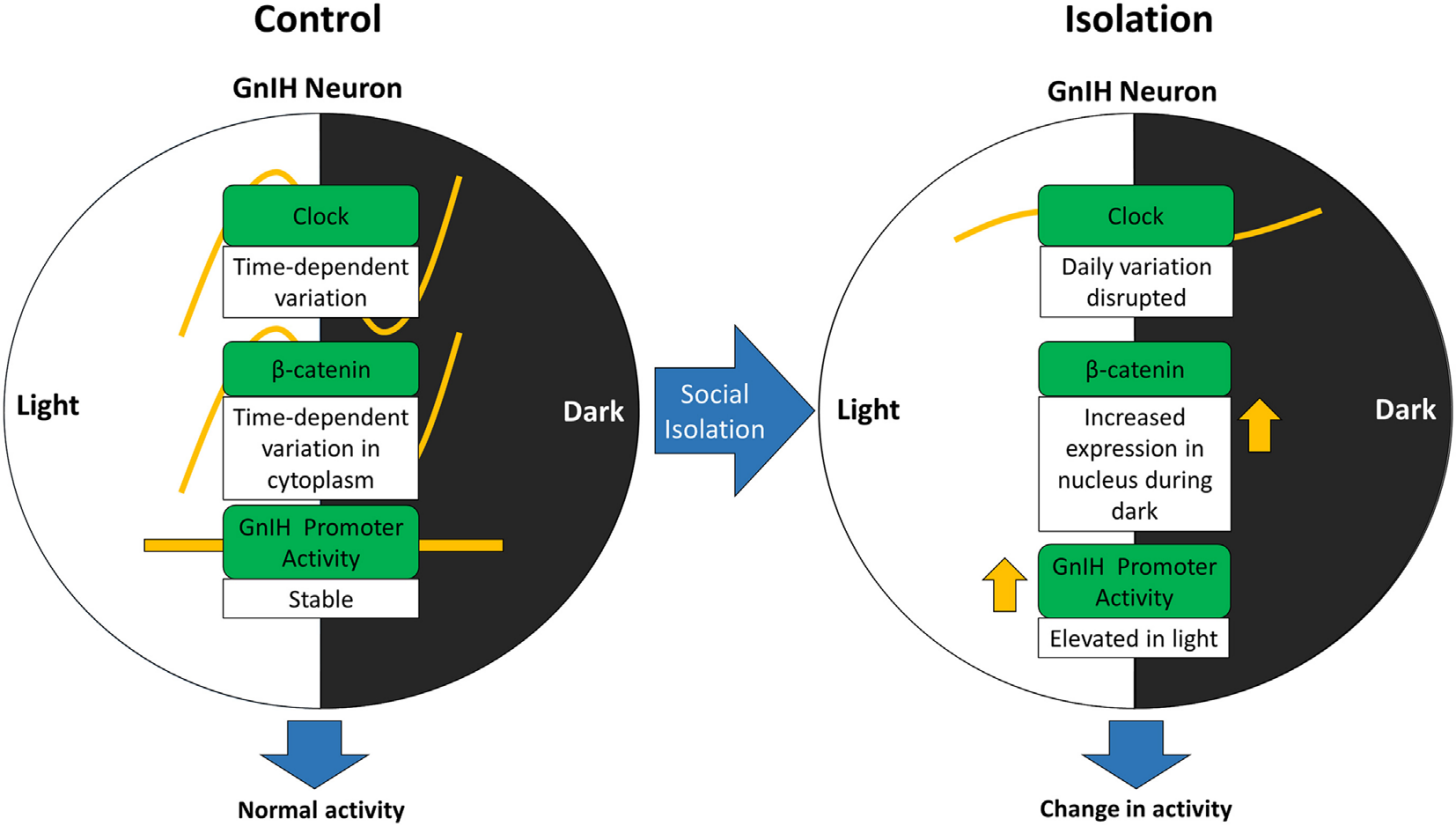
Alteration of gonadotropin-inhibitory hormone (GnIH) neuronal activity may involve change in β-catenin activity due to disturbances in CLOCK expression stemming from social isolation.

We propose that β-catenin activity can be altered due to changes in CLOCK expression stemming from social isolation and that this signaling might be regulated by social stress (Figure 7). The increase in nuclear β-catenin translocation in the GnIH neurons of isolated rats indicates that social isolation may affect β-catenin in its capacity as a transcription factor within those neurons. To address this, we plan to use luciferase assays to investigate the changes in β-catenin expression in relation to inducible GnIH promoter activity, in the future.

## Conclusion

In this study, we demonstrated the colocalization of CLOCK and β-catenin in GnIH neurons of the DMH region. We also showed that social isolation appears to invert CLOCK expression patterns in GnIH neurons across time. Furthermore, we also noted the effect of social isolation on modifying the expression of cytoplasmic β-catenin in GnIH neurons, which could be associated with heightened GnIH promoter activity in the light phase *via* measuring EGFP fluorescent intensity. Given that we also noted elevated nuclear translocation of β-catenin in isolated rodents during the dark phase, it is possible that CLOCK, β-catenin, and GnIH are connected in a pathway that culminates in the control of GnIH neuronal activity. As GnIH has been indicated to have a role in stress, the role of CLOCK and β-catenin in concert with GnIH neuronal activity may point toward a circadian component in the maintenance of regular neuronal activity under chronically stressful conditions, such as that induced by social isolation. Our findings continue to support the long-held consensus on the negative effects of social isolation and provide new insights into circadian regulation of GnIH neuronal activity.

## Ethics Statement

Animal welfare and experimental ethics in our institute. SPF animal facility was followed in line with the authorized guidelines laid out by Monash University Animal Ethics Community (MARP/2012/140, MARP/2017/021).

## Author Contributions

TS and IP designed this research. CT conducted all experiments and did analysis together with TS and IP. CT prepared a manuscript and TS and IP edited it.

## Conflict of Interest Statement

The authors declare that the research was conducted in the absence of any commercial or financial relationships that could be construed as a potential conflict of interest.

## Supplementary Material

Figure S1 Specificity test for CLOCK and β-catenin antibodies. Blank buffer test was conducted for CLOCK and β-catenin. **(A)** Result of CLOCK-immunostaining. **(B)** The result of the same immunostaining process with the CLOCK antibody absent. **(C)** Demonstrates β-catenin antibody staining, **(D)** with the antibody absent and **(E)** with the peptide absorption test. Scale bar = 50 µm.

## References

[1] Holt-Lunstad J Smith TB Layton JB. Social relationships and mortality risk: a meta-analytic review. PLoS Med (2010) 7(7):e1000316. 10.1371/journal.pmed.1000316 20668659 PMC2910600

[2] House JS Landis KR Umberson D Social relationships and health. Science (1988) 241(4865):540 10.1126/science.3399889 3399889

[3] Koyama A Miyake Y Kawakami N Tsuchiya M Tachimori H Takeshima T Lifetime prevalence, psychiatric comorbidity and demographic correlates of ‘hikikomori’ in a community population in Japan. Psychiatry Res (2010) 176:69–74. 10.1016/j.psychres.2008.10.019 20074814

[4] Naoji K Motohiro S Yasukazu K Yoshikazu K Yuji K Mie K General condition of hikikomori (prolonged social withdrawal) in Japan: psychiatric diagnosis and outcome in mental health welfare centres. Int J Soc Psychiatry (2011) 59(1):79–86. 10.1177/0020764011423611 22094722

[5] Matthews T Danese A Wertz J Ambler A Kelly M Diver A Social isolation and mental health at primary and secondary school entry: a longitudinal cohort study. J Am Acad Child Adolesc Psychiatry (2014) 54(3):225–32. 10.1016/j.jaac.2014.12.008 25721188 PMC4733108

[6] Fulford AJ Marsden CA. An intact dopaminergic system is required for context-conditioned release of 5-HT in the nucleus accumbens of postweaning isolation-reared rats. Neuroscience (2007) 149(2):392–400. 10.1016/j.neuroscience.2007.08.002 17869434

[7] Lukkes JL Engelman GH Zelin NS Hale MW Lowry CRA. Post-weaning social isolation of female rats, anxiety-related behavior, and serotonergic systems. Brain Res (2012) 1443:1–17. 10.1016/j.brainres.2012.01.005 22297173 PMC3294258

[8] Muchimapura S Fulford AJ Mason R Marsden CA. Isolation rearing in the rat disrupts the hippocampal response to stress. Neuroscience (2002) 112(3):697–705. 10.1016/S0306-4522(02)00107-0 12074911

[9] Clarke IJ Sari IP Qi Y Smith JT Parkington HC Ubuka T Potent action of RFamide-related peptide-3 on pituitary gonadotropes indicative of a hypophysiotropic role in the negative regulation of gonadotropin secretion. Endocrinology (2008) 149:5811–21. 10.1210/en.2008-0575 18617613

[10] Fukusumi S Yoshida H Fujii R Maruyama M Komatsu H Habata Y A new peptidic ligand and its receptor regulating adrenal function in rats. J Biol Chem (2003) 278:46387–95. 10.1074/jbc.M305270200 12960173

[11] Kriegsfeld LJ Mei DF Bentley GE Ubuka T Mason AO Inoue K Identification and characterization of a gonadotropin-inhibitory system in the brains of mammals. Proc Natl Acad Sci U S A (2006) 103:2410–5. 10.1073/pnas.0511003103 16467147 PMC1413747

[12] Ubuka T Morgan K Pawson AJ Osugi T Chowdury VS Minakata H Identification of human GnIH homologs, RFRP-1 and RFRP-3, and the cognate receptor, GPR147 in the human hypothalamic pituitary axis. PLoS One (2009) 4:e8400. 10.1371/journal.pone.0008400 20027225 PMC2791420

[13] Ukena K Iwakoshi E Minakata K Tsutsui K. A novel rat hypothalamic RFamide-related peptide identified by immunoaffinity chromatography and mass spectrometry. FEBS Lett (2002) 512:255–8. 10.1016/S0014-5793(02)02275-5 11852091

[14] Tsutsui K Saigoh E Ukena K Teranishi H Fujisawa Y Kikuchi M A novel avian hypothalamic peptide inhibiting gonadotropin release. Biochem Biophys Res Commun (2000) 275:661–7. 10.1006/bbrc.2000.3350 10964719

[15] Kaewwongse M Takayanagi Y Onaka T. Effects of RFamide-related peptide (RFRP)-1 and RFRP-3 on oxytocin release and anxiety-related behaviour in rats. Neuroendocrinology (2011) 23:20–7. 10.1111/j.1365-2826.2010.02077.x 21029217

[16] Kirby ED Geraghty AC Ubuka T Bentley GE Kaufer D. Stress increases putative gonadotropin inhibitory hormone and decreases luteinizing hormone in male rats. Proc Natl Acad Sci U S A (2009) 106:11324–9. 10.1073/pnas.0901176106 19541621 PMC2698887

[17] Gojska NM Belsham DD. Glucocorticoid receptor-mediated regulation of Rfrp (GnIH) and Gpr147 (GnIH-R) synthesis in immortalized hypothalamic neurons. Mol Cell Endocrinol (2014) 384:23–21. 10.1016/j.mce.2013.12.015 24412804

[18] Son YL Ubuka T Narihiro M Fukuda Y Hasunuma I Yamamoto K Molecular basis for the activation of gonadotropin-inhibitory hormone gene transcription by corticosterone. Endocrinology (2012) 155(5):1817–26. 10.1210/en.2013-2076 24552400

[19] Soga T Dalpatadu SL Wong DW Parhar IS. Neonatal dexamethasone exposure down-regulates GnRH expression through the GnIH pathway in female mice. Neuroscience (2012) 218:56–64. 10.1016/j.neuroscience.2012.05.023 22626647

[20] Serra M Pisu MG Floris I Biggio G. Social isolation-induced changes in the hypothalamic-pituitary-adrenal axis in the rat. Stress (2005) 8:259–64. 10.1080/10253890500495244 16423714

[21] Butler TR Olusegun JA Weiner JL. The impact of social isolation on HPA axis function, anxiety-like behaviors, and ethanol drinking. Front Integr Neurosci (2014) 7:102. 10.3389/fnint.2013.00102 24427122 PMC3877772

[22] Soga T Wong DW Clarke IJ Parhar IS. Citalopram (antidepressant) administration causes sexual dysfunction in male mice through RF-amide related peptide in the dorsomedial hypothalamus. Neuropharmacology (2010) 59(1):77–85. 10.1016/j.neuropharm.2010.03.018 20381503

[23] Soga T Teo CH Cham KL Mohd Idris M Parhar IS. Early-life social isolation impairs the gonadotropin-inhibitory hormone neuronal activity and serotonergic system in male rats. Front Endocrinol (2015) 6:172. 10.3389/fendo.2015.00172 26617573 PMC4639717

[24] Chandrashekaran MK Marimuthu G Geetha L. Correlations between sleep and wake in internally synchronized and desynchronized circadian rhythms in humans under prolonged isolation. J Biol Rhythms (1997) 12(1):26–33. 10.1177/074873049701200105 9104688

[25] Salgado-Delgado R Osorio AT Saderi N Escobar C. Disruption of circadian rhythms: a crucial factor in the etiology of depression. Depress Res Treat (2011) 2011:1–9. 10.1155/2011/839743 21845223 PMC3154570

[26] Prosser RA. Serotonin phase-shifts the mouse suprachiasmatic circadian clock in vitro. Brain Res (2003) 966(1):110–5. 10.1016/S0006-8993(02)04206-3 12646314

[27] Prosser RA Miller JD Heller HC. A serotonin agonist phase-shifts the circadian clock in the suprachiasmatic nuclei in vitro. Brain Res (1990) 534(1–2):336–9. 10.1016/0006-8993(90)90153-3 2073598

[28] Boden MJ Varcoe TJ Kennaway DJ. Circadian regulation of reproduction: from gamete to offspring. Prog Biophys Mol Biol (2013) 113:387–97. 10.1016/j.pbiomolbio.2013.01.003 23380455

[29] Wyse CA Coogan AN. Impact of aging on diurnal expression patterns of CLOCK and BMAL1 in the mouse brain. Brain Res (2010) 1337(2010):21–31. 10.1016/j.brainres.2010.03.113 20382135

[30] Mason AO Duffy S Zhao S Ubuka T Bentley GE Tsutsui K Photoperiod and reproductive condition are associated with changes in RFamide-related peptide (RFRP) expression in Syrian hamsters (*Mesocricetus auratus*). J Biol Rhythms (2010) 25:176–85. 10.1177/0748730410368821 20484689 PMC3266107

[31] Ubuka T Inoue K Fukuda Y Mizuno T Ukena K Kriegsfeld LJ Identification, expression, and physiological functions of Siberian hamster gonadotropin-inhibitory hormone. Endocrinology (2012) 153:373–85. 10.1210/en.2011-1110 22045661 PMC3249677

[32] Chowdury VS Yamamoto K Ubuka T Bentley GE Hattori A Tsutsui K. Melatonin stimulates the release of gonadotropin-inhibitory hormone by the avian hypothalamus. Endocrinology (2010) 151:271–80. 10.1210/en.2009-0908 19952272

[33] Russo KA La JL Stephens SBZ Poling MC Padgaonkar NA Jennings KJ Circadian control of the female reproductive axis through gated responsiveness of the RFRP-3 system to VIP signalling. Endocrinology (2015) 156(7):2608–18. 10.1210/en.2014-1762 25872006 PMC4475714

[34] Yang X Wood PA Ansell CM Ohmori M Oh EY Xiong Y Beta-catenin induces beta-TrCP-mediated PER2 degradation altering circadian clock gene expression in intestinal mucosa of ApcMin/+ mice. J Biochem (2009) 145(3):289–97. 10.1093/jb/mvn167 19106159

[35] Lin F Chen Y Li X Zhao Q Tan Z Over-expression of circadian clock gene Bmal1 affects proliferation and the canonical Wnt pathway in NIH-3T3 cells. Cell Biochem Funct (2012) 31(2):166–72. 10.1002/cbf.2871 22961668

[36] Behrens J von Kries JP Kuhl M Bruhn L Wedlich D Grosschedl R Functional interaction of β-catenin with the transcription factor LEF-1. Nature (1996) 382:638–42. 10.1038/382638a0 8757136

[37] Huber O Korn R McLaughlin J Ohsugi M Hermann BG Kemler R Nuclear localization of β-catenin by interaction with transcription factor LEF-1. Mech Dev (1996) 59:3–10. 10.1016/0925-4773(96)00597-7 8892228

[38] Takeichi M Cadherins: a molecular family important in selective cell-cell adhesion. Annu Rev Biochem (1989) 1990(59):237–52. 10.1146/annurev.bi.59.070190.001321 2197976

[39] Huelsken J Behrens J The Wnt signalling pathway. J Cell Sci (2002) 115:3977–8. 10.1242/jcs.00089 12356903

[40] Karege F Perroud N Burkhardt S Fernandez R Ballmann E La Harpe R Protein levels of β-catenin and activation state of glycogen synthase kinase-3β in major depression. A study with postmortem prefrontal cortex. J Affect Disord (2012) 136(2012):185–8. 10.1016/j.jad.2011.09.024 22036797

[41] Dias C Feng J Sun H Shao NY Mazei-Robison MS Damez-Werno D β-catenin mediates stress resilience through Dicer1/microRNA regulation. Nature (2014) 516:51–5. 10.1038/nature13976 25383518 PMC4257892

[42] Soga T Kitahashi T Clarke IJ Parhar IS. Gonadotropin-inhibitory hormone promoter-driven enhanced green fluorescent protein expression decreases during aging in female rats. Endocrinology (2014) 155:1944–55. 10.1210/en.2013-1786 24605826

[43] Falcon E Ozburn A Mukherjee S Roybal K McClung CA. Differential regulation of the period genes in striatal regions following cocaine exposure. PLoS One (2013) 8(6):e66438. 10.1371/journal.pone.0066438 23776671 PMC3679086

[44] Xu H Gustafson CL Sammons PJ Khan SK Parsley NC Ramanathan C Cryptochrome 1 regulates the circadian clock through dynamic interactions with the BMAL1 C terminus. Nat Struct Mol Biol (2015) 22(6):476–84. 10.1038/nsmb.3018 25961797 PMC4456216

[45] Dardente H Birnie M Lincoln GA Hazlerigg DG. RFamide-related peptide and its cognate receptor in the sheep: cDNA cloning, mRNA distribution in the hypothalamus and the effect of photoperiod. J Neuroendocrinol (2008) 20:1252–9. 10.1111/j.1365-2826.2008.01784.x 18752651

[46] Li JZ Bunney BG Meng F Hagenauer MH Walsh DM Vawter MP Circadian patterns of gene expression in the human brain and disruption in major depressive disorder. Proc Natl Acad Sci U S A (2013) 110(24):9950–5. 10.1073/pnas.1305814110 23671070 PMC3683716

[47] Zhu Z Hua B Xu L Yuan G Li E Li X CLOCK promotes 3T3-L1 cell proliferation via Wnt signaling. IUBMB Life (2016) 68(7):557–68. 10.1002/iub.1512 27194636

[48] Dunand-Sauthier I Santiago-Raber ML Capponi L Vejnar CE Schaad O Irla M Silencing of c-Fos expression by microRNA-155 is critical for dendritic cell maturation and function. Blood (2011) 117(17):4490–500. 10.1182/blood-2010-09-308064 21385848

[49] Vesely PW Staber PB Hoefler G Kenner L. Translational regulation mechanisms of AP-1 proteins. Mutat Res (2009) 682(1):7–12. 10.1016/j.mrrev.2009.01.001 19167516

[50] Lu T Aron L Zullo J Pan Y Kim H Chen Y REST and stress resistance in ageing and Alzheimer’s disease. Nature (2014) 507:448–54. 10.1038/nature13163 24670762 PMC4110979

