# Supplementary material for: Social Isolation Modulates CLOCK Protein and Beta-Catenin Expression Pattern in Gonadotropin-Inhibitory Hormone Neurons in Male Rats
Source: Front Endocrinol (Lausanne). 2017 Sep 7;8:225. doi: 10.3389/fendo.2017.00225 (PMC5594079; doi:10.3389/fendo.2017.00225)
Supplement: Supplementary file 1 [file Table_1.docx]

**Supplemental Table 1: Specifics for CLOCK and β-catenin antibodies.** The table details the source, concentration used and the immunogen sequence that the antibodies bind to.


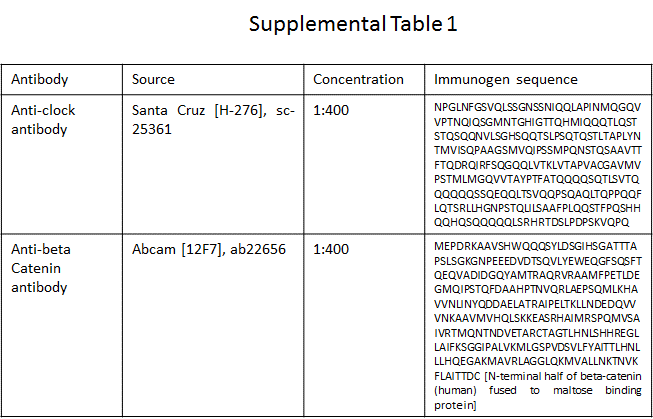


The clock antibody used in this experiment has been discontinued and the company no longer provides the appropriate peptide, however the antibody has been tested successfully in previous studies. ([Falcon et al., 2013](#_ENREF_10); [Xu et al., 2015](#_ENREF_48))
