# Supplementary figures and images for: Social Isolation Modulates CLOCK Protein and Beta-Catenin Expression Pattern in Gonadotropin-Inhibitory Hormone Neurons in Male Rats
Source: Front Endocrinol (Lausanne). 2017 Sep 7;8:225. doi: 10.3389/fendo.2017.00225 (PMC5594079; doi:10.3389/fendo.2017.00225)

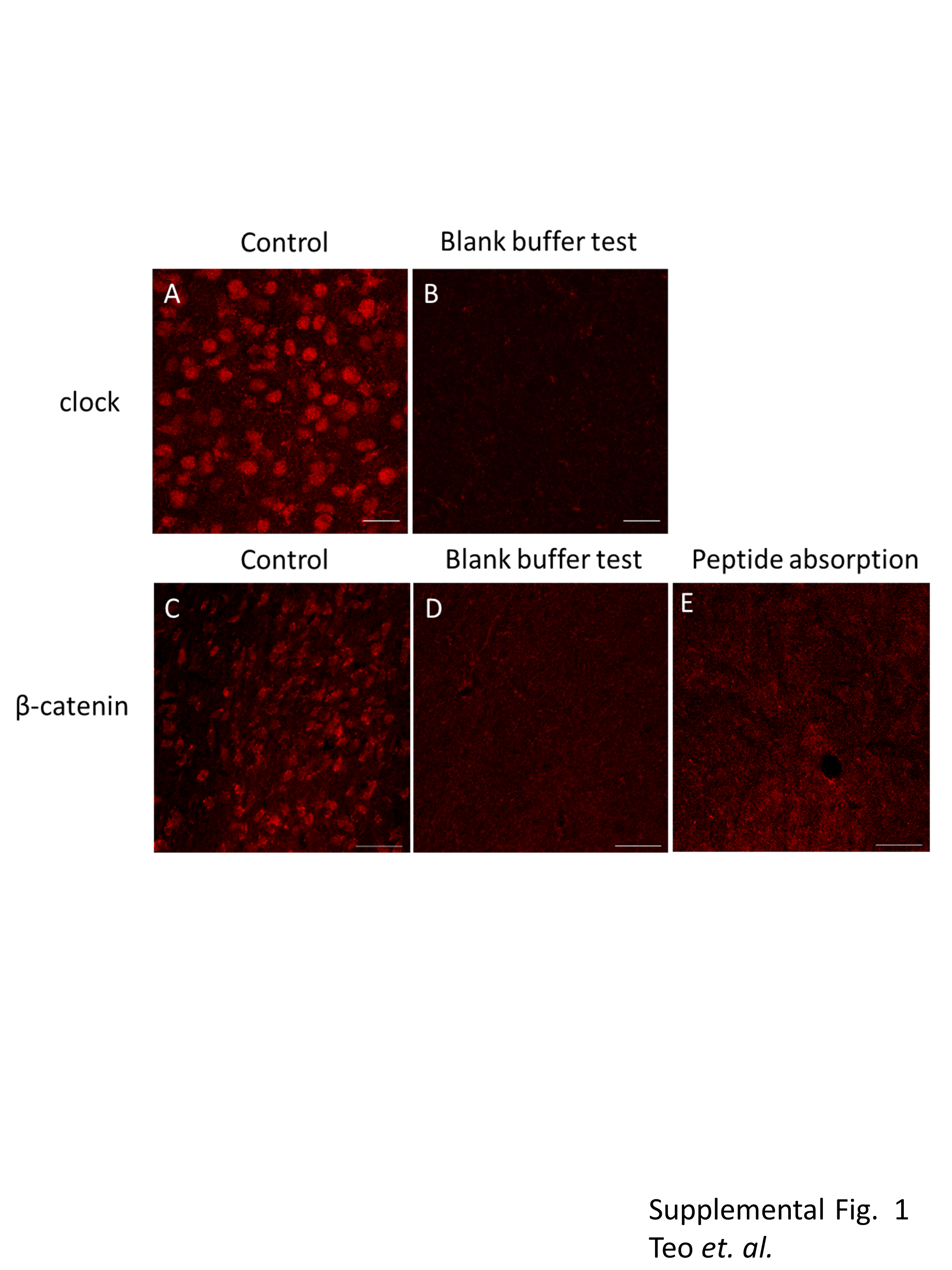

Supplement: Figure S1 — Specificity test for CLOCK and β-catenin antibodies. Blank buffer test was conducted for CLOCK and β-catenin. (A) Result of CLOCK-immunostaining. (B) The result of the same immunostaining process with the CLOCK antibody absent. (C) Demonstrates β-catenin antibody staining, (D) with the antibody absent and (E) with the peptide absorption test. Scale bar = 50 µm. [file Image_1.TIF]
